# Cited4 is related to cardiogenic induction and maintenance of proliferation capacity of embryonic stem cell-derived cardiomyocytes during *in vitro* cardiogenesis

**DOI:** 10.1371/journal.pone.0183225

**Published:** 2017-08-17

**Authors:** Junichiro Miake, Tomomi Notsu, Katsumi Higaki, Kyoko Hidaka, Takayuki Morisaki, Kazuhiro Yamamoto, Ichiro Hisatome

**Affiliations:** 1 Department of Molecular Medicine and Therapeutics, Faculty of Medicine, Tottori University, Yonago, Japan; 2 Department of Genetic Medicine and Regenerative Therapeutics, Institute of Regenerative Medicine and Biofunction, Tottori University, Yonago, Japan; 3 Research Center for Bioscience and Technology, Tottori University, Yonago, Japan; 4 Center for Fundamental Education, The University of Kitakyushu, Kitakyushu, Japan; 5 Department of Clinical Engineering, School of Health Sciences, Tokyo University of Technology, Ota, Japan; Georgia Regents University, UNITED STATES

## Abstract

Cardiac progenitor cells have a limited proliferative capacity. The CREB-binding protein/p300-interacting transactivator, with the Glu/Asp-rich carboxy-terminal domain (Cited) gene family, regulates gene transcription. Increased expression of the *Cited4* gene in an adult mouse is associated with exercise-induced cardiomyocyte hypertrophy and proliferation. However, the expression patterns and functional roles of the *Cited4* gene during cardiogenesis are largely unknown. Therefore, in the present study, we investigated the expression patterns and functional roles of the *Cited4* gene during *in vitro* cardiogenesis. Using embryoid bodies formed from mouse embryonic stem cells, we evaluated the expression patterns of the *Cited4* gene by quantitative reverse transcriptase-polymerase chain reaction. *Cited4* gene expression levels increased and decreased during the early and late phases of cardiogenesis, respectively. Moreover, *Cited4* gene levels were significantly high in the cardiac progenitor cell population. A functional assay of the *Cited4* gene in cardiac progenitor cells using flow cytometry indicated that overexpression of the *Cited4* gene significantly increased the cardiac progenitor cell population compared with the control and knockdown groups. A cell proliferation assay, with 5-ethynyl-2′-deoxyuridine incorporation and Ki67 expression during the late phase of cardiogenesis, indicated that the number of troponin T-positive embryonic stem cell-direived cardiomyocytes with proliferative capacity was significantly greater in the overexpression group than in the control and knockdown groups. Our study results suggest that the *Cited4* gene is related to cardiac differentiation and maintenance of proliferation capacity of embryonic stem cell-derived cardiomyocytes during *in vitro* cardiogenesis. Therefore, manipulation of *Cited4* gene expression may be of great interest for cardiac regeneration.

## Introduction

*Cited4* is a gene of the CREB-binding protein/p300-interacting transactivator, with Glu/Asp-rich carboxy-terminal domain (Cited) family and regulates gene transcription [1]. The *Cited4* gene is expressed in the developing heart and the expression is restricted to the endocardium [1]. In the adult mouse, the increased expression of the *Cited4* with exercise is associated with cardiomyocyte hypertrophy and proliferation [2].

Embryonic stem (ES) cell-derived cardiogenesis using embryoid bodies (EBs) formed from mouse ES cells is a useful *in vitro* system to assess the molecular mechanisms of cardiogenesis [3,4]. There is an increased need to understand the biological properties of cardiac progenitor cells for their application in regenerative medicine. Studies of *in vitro* cardiogenesis suggest that the proliferative capacity of ES cell-derived cardiomyocytes is markedly decreased after cardiogenic induction [5].

During cardiogenesis, undifferentiated pluripotent stem cells give rise to early mesodermal cells, lateral mesodermal cells, and then cardiac progenitor cells. *Rex1* [6], *Bra* [7], *Flk1* [8], and *Nkx2*.*5* [9,10] are lineage markers for undifferentiated pluripotent stem cells, early mesodermal cells, lateral mesodermal cells, and cardiac progenitor cells, respectively. However, the expression patterns and the functional roles of the *Cited4* gene during *in vitro* cardiogenesis are largely unknown.

In this study, we aimed to investigate the time-dependent expression patterns of the *Cited4* in the EBs, compare the lineage-specific expressions of the *Cited4*, and investigate whether the *Cited4* is associated with cardiogenic induction and proliferation capacity of ES cell-derived cardiomyocytes during *in vitro* cardiogenesis.

## Materials and methods

### Culture of mouse embryonic stem cells and *in vitro* cardiogenesis

The 129/Ola-derived ES cell lines used in this study are ht7, which was provided by Hitoshi Niwa, Kumamoto University, Japan, and its derivatives. The ht7 carries a hygromycin resistance gene in one of the Oct-3/4 loci, which allows selection of Oct-3/4-positive undifferentiated stem cells [11]. Hcgp7 has been previously reported: hcgp7 cells are derived from ht7 cells and carry a GFP reporter gene in one of the Nkx2.5 loci [12]. ES cells were maintained on gelatin-coated dishes without feeder cells in Glasgow Minimum Essential Medium (Sigma-Aldrich, St. Louis, MO, USA) supplemented with 10% fetal bovine serum (JRH Bioscience, Lenexa, KS, USA), non-essential amino acids (Gibco-BRL; Life Technologies, Carlsbad, CA, USA), 1 mmol/L sodium pyruvate (Sigma-Aldrich), penicillin-streptomycin-glutamine (Gibco-BRL), 0.1 mmol/L 2-mercaptoethanol (Sigma-Aldrich), 1000 units/mL leukaemia inhibitory factor (LIF, Chemicon; Millipore, Billerica, MA, USA), and 0.1 mg/mL Hygromycin B (Gibco-BRL). For cardiac differentiation, 500 ES cells in 20 μL aliquots of differentiation medium (maintenance medium without LIF and Hygromycin) were cultured in hanging drops for 3 days. After that, the EBs were further cultured in a floating condition. To evaluate the cardiac differentiation efficiency of ES cells, some of the resultant EBs were transferred to individual wells of gelatin-coated 24-well culture plates on the fifth day, and the 24-well culture plates were monitored every day under a microscope to detect the appearance of spontaneously contracting cardiomyocytes. The percentage of EBs exhibiting spontaneous contraction was calculated as the cardiac differentiation efficiency. The medium was changed every other day. The day when hanging drop culture was initiated was defined as day 1.

### Quantitative reverse transcriptase-polymerase chain reaction

Total RNA extraction from ES cells before differentiation, and EBs on day 3.5, 4.5, 5.5, 6.5, 7.5, 8.5, 9.5, and 10.5 after differentiation, was performed using an RNeasy Mini Kit (Qiagen, Valencia, CA, USA). First-strand cDNA synthesis was performed using SuperScript II reverse transcriptase (Gibco-BRL). Quantitative reverse transcriptase-polymerase chain reaction (qRT-PCR) was performed with a LightCycler SYBR Green I Kit (Roche Diagnostics, Mannheim, Germany) according to the manufacturer’s protocol. Gene expression levels were determined by a LightCycler 1.2 (Roche, Basel, Switzerland). The qRT-PCR data were analyzed using the second derivative maximum method available with LightCycler Software Version 3.5.3. The sequences of the qRT-PCR primers for *Rex1*, *Brachyury* (*Bra*), *Flk1*, *Nkx2*.*5*, *Cited4*, and *β-actin* genes are listed in Table 1. The *β-actin* gene was used as a reference molecule.

**Table 1:** Sequence of the primers for qRT-PCR.

Gene Symbol	Accession No.		Primer sequence	Product length (bp)
**Rex1**	NM_009556	Forward primer	5'-TCGAGTCTTCTTTGGAGTAC-3'	152
		Reverse primer	5'-TAAGACACCACAGTACACAC-3'	
**Bra**	NM_009309	Forward primer	5'-CATTACACACCACTGACGCA-3'	165
		Reverse primer	5'-CATAGATGGGGGTGACACAG-3'	
**Flk1**	NM_010612	Forward primer	5'-ATGACAGCCAGACAGACAGT-3'	188
		Reverse primer	5'-GGTGTCTGTGTCATCTGAGT-3'	
**Nkx2.5**	NM_008700	Forward primer	5'-CAAGTGCTCTCCTGCTTTCC-3'	136
		Reverse primer	5'-GGCTTTGTCCAGCTCCACT-3'	
**Cited4**	NM_019563	Forward primer	5'-ACCGAGCTCATCGACGAAG-3'	164
		Reverse primer	5'-GGTGGCTCTCAACAGCTCAC-3'	
**β-actin**	X03672	Forward primer	5'-CAACCGTGAAAAGATGAC-3'	238
		Reverse primer	5'-CAGGATCTTCATGAGGTAGT-3'	

### Plasmid construction and transfection

Enhanced green fluorescent protein (EGFP) was used as a reporter of expression. The Rex1-promoter EGFP construct was a gift from Dr. Yasuaki Shirayoshi. Briefly, the Rex1-promoter EGFP construct was generated by cloning a promoter fragment of the mouse *Rex1* gene ranging from −270 to +51 of the 5′ region of *Rex1* into pd2EGFP-1 (Clontech, Mountain View, CA, USA). The Bra-promoter EGFP construct was generated by cloning a promoter fragment of the mouse *Bra* gene ranging from −484 to +134 of the 5′ region of *Bra* into the promoter-less EGFP reporter vector, pEGFP-1 (Invitrogen; Life Technologies). The *Bra* promoter region of the plasmid TpGL3 (a gift from Dr. Rolf Kemler) was digested by Sac I and isolated by electrophoresis, and then ligated into the pEGFP-1 plasmid.

The *Cited4* cDNA fragment was obtained by RT-PCR with total RNA extracted from the ht7 cell-derived EBs on day 7.5. Primers for cloning the *Cited4* gene are presented in Table 2. Amplified *Cited4* gene fragments were ligated into a pGEM-T vector (Promega, Madison, WI, USA). The *Cited4* gene fragments were then digested from the plasmid by Eco RI and Spe I, and isolated by electrophoresis. To create p3xFLAG-CMV-10-Cited4 plasmid (pCited4), the digested *Cited4* gene fragments were cloned into a p3xFLAG-CMV-10 expression vector (Sigma-Aldrich) digested by Eco RI and Xba I.

**Table 2:** Sequence of the primers for cloning of Cited4.

	Primer sequence
Forward primer	5'-TATGGCCGACCACCTGATGC-3'
Reverse primer	5'-GTGGTGGCTCTCAACAGCTCAC-3'

For the RNA interference assay, a plasmid expressing double-stranded, small interfering RNA against the mouse *Cited4* gene was generated using the siLentGene U6 Hairpin Cloning System (Promega). To generate the *Cited4* knockdown vector (siCited4), the DNA cassette containing the hairpin structure to target the *Cited4* gene was amplified by PCR and inserted into the psiLentGene vector. To create the control vector (scrambled Cited4-siRNA), a nonspecific siRNA duplex containing the same nucleotides but in irregular sequence was prepared. The siRNA and scrambled siRNA sequences are provided in Table 3.

**Table 3:** Sequence of siRNA targeting Cited4.

siRNA	Sequence
**siRNA**	5'-ATCTAAAAAGTGAACGAATCCGATTTATTCTCTTGAAATAAATCGGATTCGTTCACGGTGTTTCGTCCTTTCCACAAGA-3'
**scrambled siRNA**	5'-ATCTAAAAAGATGCTGCGCTAAATTAATTCTCTTGAAATTAATTTAGCGCAGCATCGGTGTTTCGTCCTTTCCACAAGA-3'

The constructed plasmids were transfected into the ht7 or hcgp7 cells after linearizing the constructs with Lipofectamine and Plus Reagent. Approximately 24 h after transfection, the transfected cells were transferred to medium containing G418 sodium salt at a concentration of 400 μg/mL for more than 1 week to select stable transfectants. The Rex1-GFP-transfected ht7, Bra-GFP-transfected ht7, pCited4-transfected ht7 and pCited4-transfected hcgp7 cells were designated as Rex1-ht7, Bra-ht7, Cited4-ht7 and Cited4-hcgp7, respectively. The siCited4 vector was transfected into ht7 and hcgp7 cells, and designated as siCited4-ht7 and siCited4-hcgp7, respectively. Downregulation of *Cited4* gene expression by functional siRNA, but not by the control, was also confirmed by qRT-PCR.

### Flow cytometry and cell sorting

ES cells and EBs were dissociated with trypsin (0.25%)-EDTA (1 mmol/L) on the indicated differentiation day. The dissociated cells were suspended in Hank’s Balanced Salt Solution (Cambrex; Lonza, Basel, Switzerland) with 1% bovine serum albumin (BSA, Sigma-Aldrich). Phycoerythrin (PE) and GFP fluorescence was detected using a 488-nm argon laser with an EPICS^®^ ELITE ESP (Beckman Coulter, Brea, CA, USA). For sorting Rex1-positive cells, the dissociated Rex1-ht7 cells on day 0 were incubated with an anti-mouse E-cadherin antibody followed by a PE-conjugated anti-mouse IgG antibody. For sorting Bra-positive and Flk1-positive cells, the dissociated Bra-ht7 cells on day 4.5 were incubated with a PE-conjugated anti-mouse Flk1 antibody. For sorting Nkx2.5-positive cells, the dissociated hcgp7 cells on day 7.5 were incubated with the PE-conjugated anti-mouse Flk1 antibody. Propidium iodide detected dead cells. Antibodies are listed in Table 4.

**Table 4:** List of antibodies for flow cytometry.

Antibody	Dilution	Catalog Number	Company
**Anti-mouse E-cadherin (monoclonal)**	1:1000	M108	TaKaRa Bio
**PE-conjugated anti-mouse Flk1 (monoclonal)**	1:1000	555308	BD Biosciences
**PE-conjugated anti-mouse IgG**	1:1000	M30004	Molecular Probes

### Cell proliferation assay for cardiac progenitors

The cell proliferation assay was performed using 5-ethynyl-2′-deoxyuridine (EdU) incorporation and Ki67 expression. EdU incorporation was performed with a Click-iT EdU Alexa Fluor^®^ 647 Imaging Kit (Life Technologies) according to the manufacturer’s protocol. The EBs on day 7.5, 10.5, and 15.5 of *in vitro* differentiation were cultured for 1 hour with EdU. The prepared EBs were seeded onto glass coverslips, fixed in 4% paraformaldehyde and permeabilized with 0.1% Triton X-100. After blocking in 3% BSA, cells were immunocytochemically stained with antibodies. Ki67 expression was detected with an antibody against Ki67, followed by Alexa Fluor^®^ 568 goat anti-rabbit IgG antibody. Nucleic acid staining was performed with 2'-[4-ethoxyphenyl]-5-[4-methyl-1-piperazinyl]-2,5'-bi-1H-benzimidazole trihydrochloride trihydrate (Hoechst 33342). Troponin T (TnT) expression was detected with an antibody against TnT, followed by Alexa Fluor^®^ 488 goat anti-mouse IgG antibody. Images of samples were taken using the Leica TCS SP2 confocal laser scanning microscope system (Leica Microsystems). Antibodies are listed in Table 5.

**Table 5:** List of antibodies for immunofluorescence staining.

Antibody	Dilution	Catalog Number	Company
**Anti-Ki67 (polyclonal)**	1:1000	ab15580	Abcam
**Anti-Troponin T (monoclonal)**	1:1000	MA5-12960	Thermo Fisher Scientific
**Alexa Fluor^®^ 568 goat anti-rabbit IgG**	1:1000	A21069	Molecular Probes
**Alexa Fluor^®^ 488 goat anti-mouse IgG**	1:1000	A11029	Molecular Probes

### Statistical analyses

Continuous variables are presented as the mean ± standard error of the mean. Categorical variables were analyzed by chi-square analysis followed by Bonferroni’s post-hoc comparison tests. All statistical analyzes were performed with the software R (The R Foundation for Statistical Computing, Vienna, Austria; version 3.1.1). *P* < 0.05 was considered statistically significant.

## Results

### *Cited4* gene expression increases transiently during the early phase of cardiogenesis

**Fig 1:**
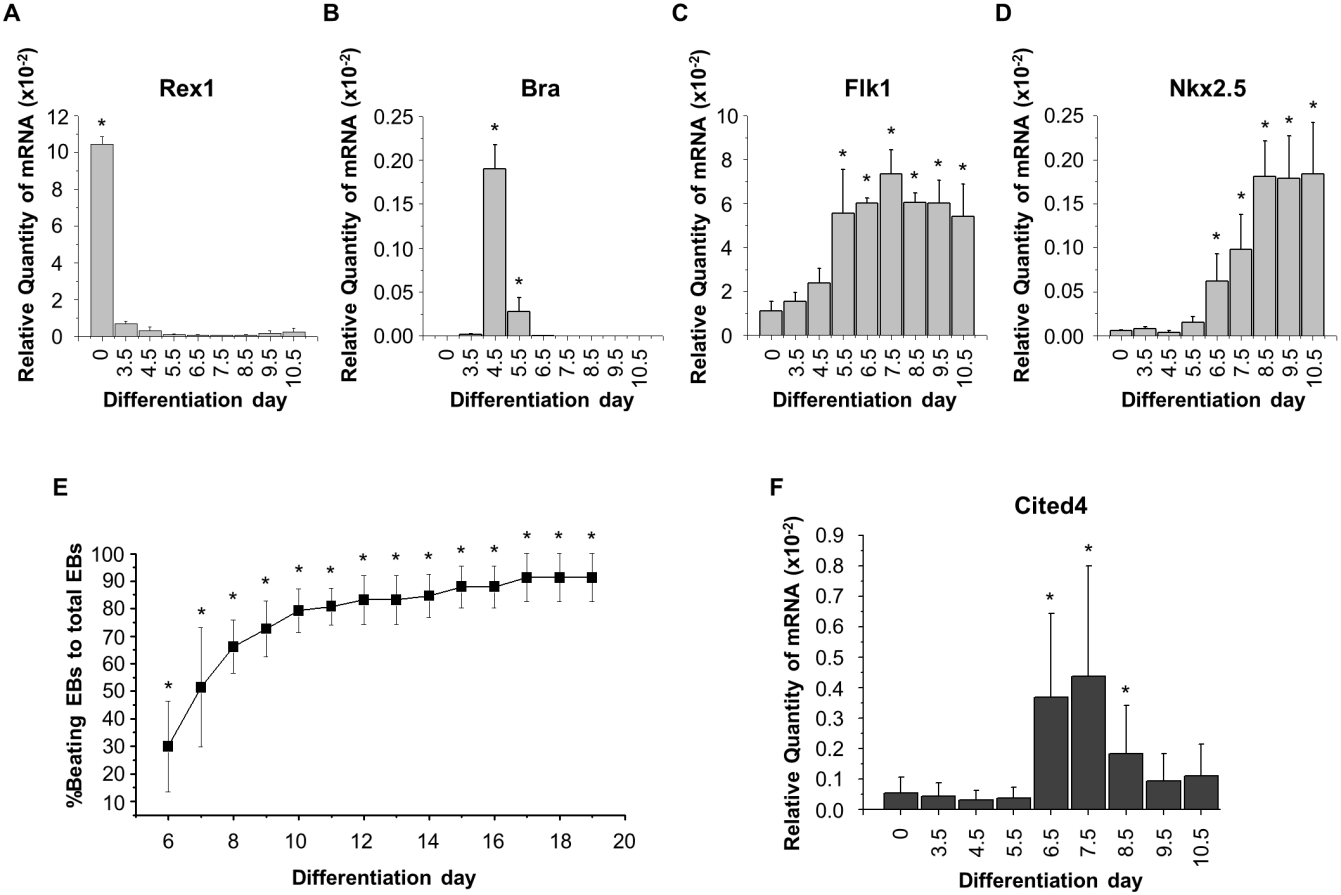
The *Cited4* gene is expressed transiently during the early cardiac stage of *in vitro* cardiogenesis. (A-D) mRNA expression levels of lineage marker genes during *in vitro* cardiogenesis were measured by qRT-PCR and normalized to *β-actin* mRNA levels. *Rex1*, *Bra*, *Flk1*, and *Nkx2*.*5* genes were lineage markers for undifferentiated pluripotent stem cells, early primitive mesodermal cells, lateral mesodermal cells, and cardiac progenitor cells, respectively (N = 4). (E) The proportion of beating EBs, a classical *in vitro* cardiogenesis marker, was evaluated during *in vitro* cardiogenesis (N = 3, n = 240). (F) mRNA expression levels of the *Cited4* gene during *in vitro* cardiogenesis were measured by qRT-PCR and normalized to *β-actin* mRNA levels (N = 3). N: number of experiments; n: total number of EBs counted. *P < 0.05 vs. *Rex1* expression at day 4.5, *Bra* expression at day 3.5, *Flk1* expression at day 0, *Nkx2*.*5* expression at day 0, *Cited4* expression at day 0, and proportion of beaing EBs at day 5.0.

To explore time-dependent expression patterns of the *Cited4* gene during *in vitro* cardiogenesis, we studied the time course of expression levels of the *Cited4* gene and lineage marker genes. We used the EBs as an *in vitro* differentiation system, which comprised cell aggregates formed with mouse ES cells to recapitulate *in vivo* cardiac differentiation. We studied the expression patterns of lineage marker genes with qRT-PCR. Consequently, *Rex1* gene expression was high at differentiation day 0 and decreased during differentiation (Fig 1A). *Bra* gene expression was transient during differentiation with a peak expression level at day 4.5 (Fig 1B). Subsequently, *Flk1* gene expression increased at day 4.5–5.5 (Fig 1C). *Nkx2*.*5* gene expression increased at day 6.5–7.5 (Fig 1D), which was consistent with the initiation of cellular beating (Fig 1E). *Cited4* gene expression was transient at the early phase of cardiogenesis; i.e. *Cited4* gene expression increased when the expression of the *Bra* and the *Nkx2*.*5* genes decreased and increased, respectively, and the EBs started beating (Fig 1F).

### The *Cited4* gene is expressed specifically in a cardiac progenitor cell population during *in vitro* cardiogenesis

**Fig 2:**
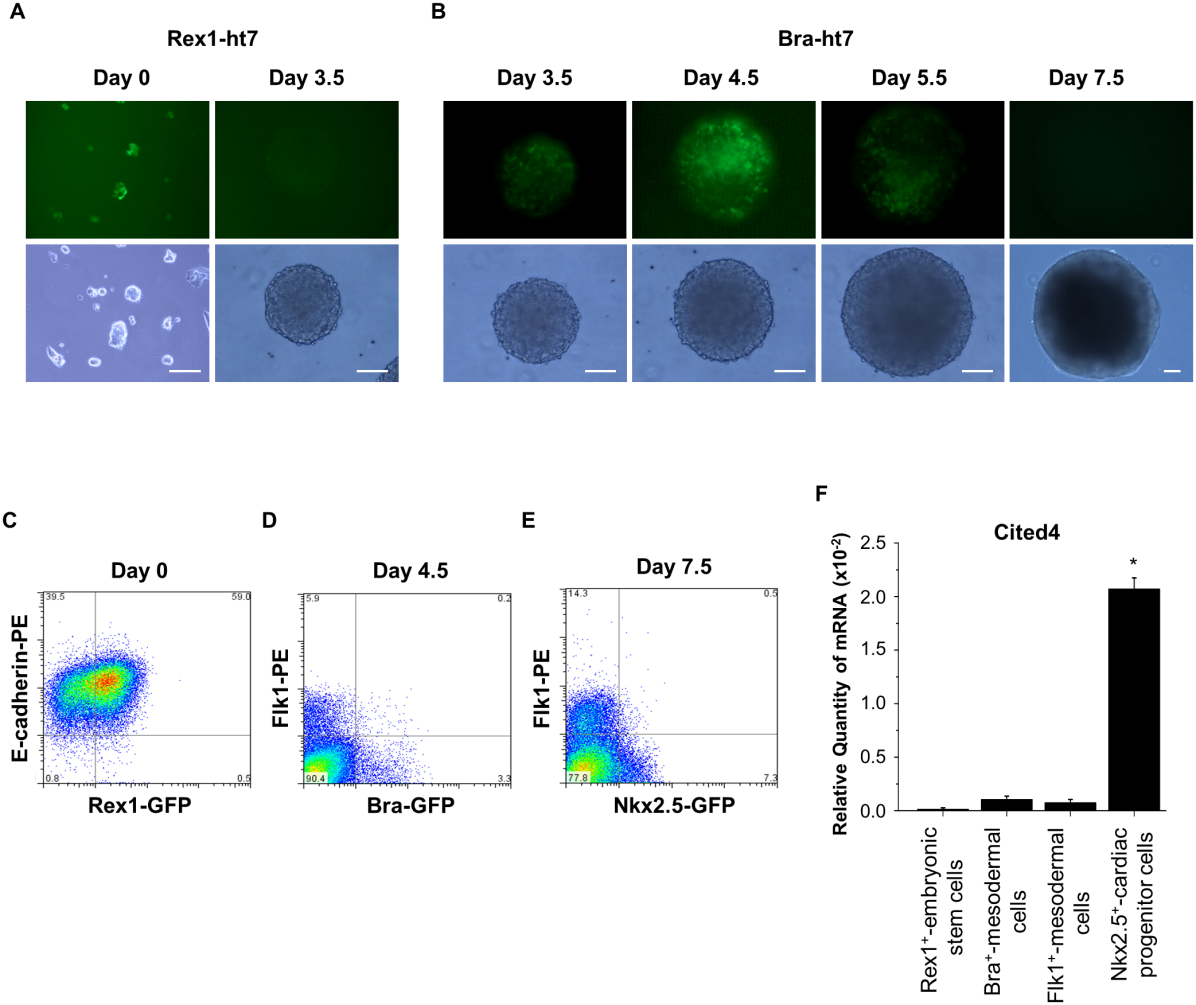
The *Cited4* gene is expressed specifically in cardiac progenitor cell populations during *in vitro* cardiogenesis. (A) Time-dependent expression of GFP under the control of a Rex1-promoter was evaluated at day 0 of undifferentiation embryonic stem cells and day 3.5 in the EBs formed with the Rex1-ht7 cell line. Scale bar = 100 μm. (B) Time-dependent expression of GFP under the control of a Bra-promoter was evaluated at day 3.5, day 4.5, day 5.5, and day 7.5 in the EBs formed with the Bra-ht7 cell line. Scale bar = 100 μm. (C) To obtain the pluripotent embryonic stem cell population, the Rex1 and E-cadherin double positive cell population was isolated from the Rex1-ht7 cell line at day 0 before forming the EBs. (D) To obtain the primitive mesodermal cell population and the early lateral mesodermal cell population, the Bra-positive and Flk1-negative cell population and the Bra-negative and Flk1-positive cell population were isolated from the Bra-ht7 cell line at day 4.5 in the EBs, respectively. (E) To obtain the cardiac progenitor cell population, the Nkx2.5-positive and Flk1-negative cell population was isolated from the hcgp7 cell line at day 7.5 in the EBs. (F) The *Cited4* gene expressions in the isolated lineage marker-positive cell populations were evaluated by qRT-PCR and normalized to *β-actin* mRNA levels (N = 3). Values are presented as the mean ± SEM. N: number of experiments. *P < 0.05 vs. Rex1-positive embrynic stem cells.

To study the lineage-specific expression of the *Cited4* gene during *in vitro* cardiogenesis, we evaluated *Cited4* gene expression in the different lineage marker-positive cell populations isolated by fluorescence-activated cell sorting. First, we confirmed that the expression patterns of GFP under the control of *Rex1* (Fig 2A) and *Bra* (Fig 2B) promoters were consistent with the expression patterns of *Rex1* and *Bra* genes as shown in Fig 1A and 1B, respectively. Then, we isolated the Rex1-GFP-positive and E-cadherin-positive undifferentiated stem cell population from the Rex1-ht7 cell line at day 0 (Fig 2C), the Bra-positive and Flk1-negative primitive mesodermal cell population from the Bra-ht7 cell line at day 4.5 (Fig 2D), the Bra-negative and Flk1-positive early lateral mesodermal cell population from the Bra-ht7 cell line at day 4.5 (Fig 2D), and the Nkx2.5-positive and Flk1-negative early cardiac progenitor cell population from the hcgp7 cell line at day 7.5 (Fig 2E). Consequently, the level of *Cited4* gene expression was specifically high in the early cardiac progenitor cell population (Fig 2F). Taken together with the results shown in Fig 1F, these results indicated that *Cited4* expression was spatiotemporally specific to the early cardiac progenitor cell population, suggesting that the *Cited4* gene is an important cardiogenesis-related factor. Therefore, we subsequently investigated the functional roles of the *Cited4* gene in cardiogenesis.

### The *Cited4* gene increases the early cardiac progenitor cell population

**Fig 3:**
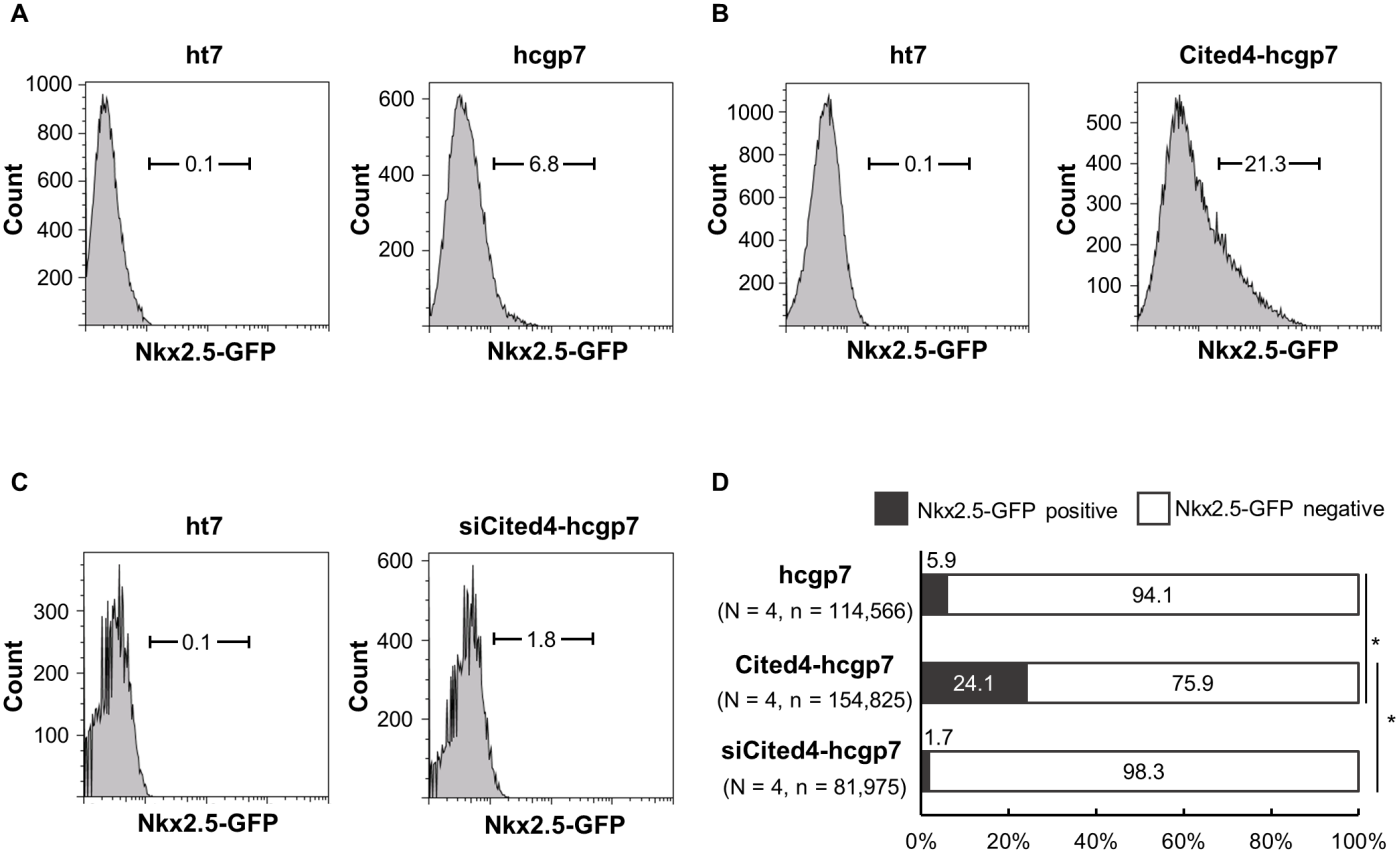
The *Cited4* gene increases the cardiac progenitor cell population. Nkx2.5-positive cardiac progenitor cells were quantified with flow cytometry at day 6.5. The ht7 cell line was used as a control for flow cytometry. The hcgp7 cell line comprised knocked-in cells with a GFP gene as a reporter at the locus of the *Nkx2*.*5* gene and was used for quantification of cardiac progenitor cells. (A) The hcgp7 cell line without modification of the *Cited4* gene was used as a control. (B) The Cited4-hcgp7 cell line comprised cells with the overexpression of the *Cited4* gene. (C) The siCited4-hcgp7 cell line comprised cells with the knockdown of the *Cited4* gene. (D) The overexpression of the *Cited4* gene significantly increased amounts of cardiac progenitor cells compared with the control and knockdown cells (*P < 0.001). N: number of experiments; n: total number of cells counted.

To study the possibility that *Cited4* gene expression levels relate to the amounts of early cardiac progenitor cell population, we created Cited4-overexpressing hcgp7 (Cited4-hcgp7) and Cited4-knockdown hcgp7 (siCited4-hcgp7) cells. Levels of the *Cited4* gene expression at day 6.5 were increased 2.0-fold and decreased 0.27-fold in the overexpressing and knockdown cells, respectively. The protein expression levels of Cited4 at day 6.5 were increased 1.7-fold and decreased 0.3-fold in the overexpressing and knockdown cells, respectively, compared with the control cells (S1 Protocol, S1 Table and S1 Fig). We then compared the amounts of the early cardiac progenitor cell populations in the EBs at day 6.5 formed from the control hcgp7 cells, the Cited4-hcgp7 cells, and the siCited4-hcgp7 cells (Fig 3A–3C). Consequently, without changing the timing of cellular beating, the Nkx2.5-positive cardiac progenitor cell population was significantly increased about 4-fold in the Cited4-hcgp7 group compared with the control group (Fig 3D); meanwhile, the Nkx2.5-positive cardiac progenitor cell population was decreased in the siCited4-hcgp7 group compared with the control group, although this did not reach statistical significance (Fig 3D). These results suggest that the *Cited4* gene is associated with the cardiogenic induction.

### The *Cited4* gene maintains the proliferation capacity of TnT-positive ES cell-derived cardiomyocytes

**Fig 4:**
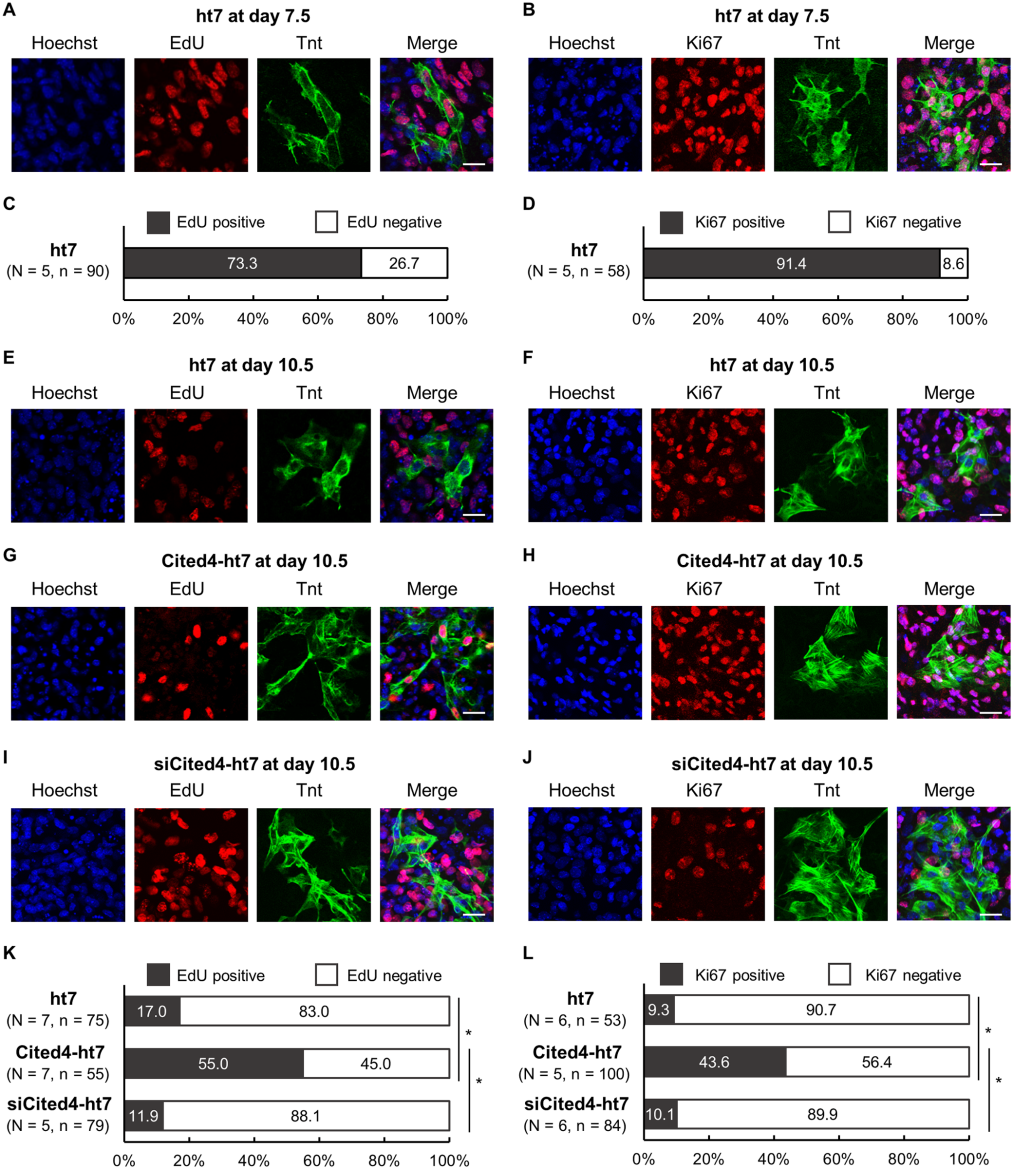
The *Cited4* gene maintains the proliferation capacity of ES cell-derived cardiomyocytes expressing the myogenic marker of TnT. The proliferation capacity of TnT-positive ES cell-derived cardiomyocytes was evaluated with EdU incorporation and Ki67 expression. (A and B) First, the proliferation capacity of TnT-positive ES cell-derived cardiomyocytes at day 7.5 was confirmed. (C and D) In the TnT-positive cardiomyocytes derived from control ht7 cells at day 7.5, 73.3% and 91.4% of the cells are EdU-positive and Ki67-positve cells, respectively. (E—J) Then, the proliferation capacities of TnT-positive cardiomyocytes derived from ht7 (E and F), Cited4-ht7 (G and H), and siCited4-ht7 (I and J) cells were compared at day 10.5. (K and L) In the TnT-positive cardiomyocytes derived from Cited4-ht7 cells at day 10.5, the EdU-positive or Ki67-positve cells were significantly increased compared with those derived from ht7 and siCited4-ht7 cells (*P < 0.001). Images were obtained with confocal laser scanning microscopy. Scale bar = 20 μm. N: number of experiments; n: total number of cells counted.

To further confirm the functional roles of *Cited4* gene in the proliferation capacity of differrentiated ES cell-derived cardiomyocytes expressing the myogenic marker of TnT, we evaluated EdU incorporation as a marker of DNA synthesis (Fig 4A, 4E, 4G and 4I) and Ki67 expression as an indicator of mitotically active cells (Fig 4B, 4F, 4H and 4J). The proliferation capacities of TnT-positive ES cell-derived cardiomyocytes derived from control ht7, Cited4-overexpressing ht7 (Cited4-ht7), and Cited4-knockdown ht7 (siCited4-ht7) cells were compared at day 10.5, at which time ES cell-derived cardiomyocytes start to decrease proliferation capacity. To do this, we first studied the proliferation capacity of TnT-positive ES cell-derived cardiomyocytes at day 7.5 from control ht7 cells (Fig 4A and 4B). As a result, 73.3% and 91.4% of the cells were EdU-positive and Ki67-positive, respectively. In the TnT-positive ES cell-derived cardiomyocytes at day 10.5, 17.0% and 9.3% of the TnT-positive ES cell-derived cardiomyocytes from control ht7 cells were EdU-positive and Ki67-positive, respectively (Fig 4E and 4F); 55.0% and 43.6% of those from Cited4-ht7 cells were EdU-positive and Ki67-positive, respectively (Fig 4G and 4H); 11.9% and 10.1% of those from siCited4-ht7 cells were EdU-positive and Ki67-positive, respectively (Fig 4I and 4J). In the TnT-positive ES cell-derived cardiomyocyte derived from Cited4-ht7 cells, the EdU-positive or Ki67-positve cells were significantly increased compared with those derived from control ht7 and siCited4-ht7 cells (Fig 4K and 4L). The proliferation competency evaluated with EdU incorporation at the extended time point of day 15.5, the proportions of EdU-positive cells among TnT-positive cells were 13.2% (15 of 114 TnT-positive cells) in the control cells, 21.7% (54 of 249 TnT-positive cells) in the overexpression cells, and 6.4% (10 of 157 TnT-positive cells) in the knockdown cells, and significant differences were observed among the three groups (*P* < 0.001 by chi-square analysis). Taken together, these results suggest that the *Cited4* gene enabled the differrentiated ES cell-derived cardiomyocytes expressing the myogenic marker of TnT to maintain the proliferation capacity during *in vitro* cardiogenesis.

## Discussion

In the present study, we identified that the *Cited4* gene expression was transient during *in vitro* cardiogenesis and specific to Nkx2.5-positive cardiac lineage cells. Furthermore, we confirmed that the *Cited4* gene was associated with cardiogenic induction and proliferation capacity of ES cell-derived cardiomyocytes expressing TnT during *in vitro* cardiogenesis.

We studied the *in vitro* expression patterns of the *Cited4* gene with those of the lineage marker genes, *Rex1*, *Bra*, *Flk1 and Nkx2*.*5*. The strong *Cited4* gene expression has been observed in developing embryonic heart with *in situ* hybridization, compared with other organs [1]. In agreement with the previous *in vivo* study, the expression of the *Cited4* gene during *in vitro* cardiogenesis in this study was specifically high in Nkx2.5-positive cardiac progenitor cells. Moreover, the expression patterns of *Rex1*, *Bra*, *Flk1 and Nkx2*.*5* during *in vitro* cardiogenesis in this study were considered to recapitulate those during *in vivo* development [6–10]. Therefore, the *in vitro* expression pattern of the *Cited4* gene together with the lineage marker genes in this study was considered to be consistent to *in vivo* mouse embryonic differentiation process. However, further investigation and comparison of the time-dependent *Cited4* expression levels in *in vitro* and *in vivo* differentiation processes are necessary.

Although, in the adult mouse, the expression of the *Cited4* is associated with exercise-induced cardiomyocyte proliferation [2], functional roles of the *Cited4* gene during cardiogenesis are largely unknown. In this study, the *Cited4* expression was spatiotemporally specific to the early cardiac progenitor cell population. Furthermore, the *Cited4* gene increased the early cardiac progenitor cell population. Moreover, we have observed that the expression levels of *Nkx2*.*5* and *Gata4* gene were increased and decreased in the *Cited4* overexpression and downregulation, respectively, compared with the control cells. Therefore, although the precise regulatory relationship between *Cited4* and *Nkx2*.*5* gene and between *Cited4* and *Gata4* gene, *Cited4* gene was suggested to be associated with the cardiogenic induction.

So far, knockouts of individual or a combination of positive cell cycle regulators, such as cyclins and cyclin-dependent kinases, failed to show a significant defect of cell proliferation in early cardiogenesis [13–17]. Cardiac progenitor cells at the early stage of *in vitro* cardiogenesis are lacking of TnT expression, and TnT has been considered to be a marker for differentiated ES cell-derived cardiomyocytes at the stage of cardiomyogenesis [4], at which stage the ES cell-derived cardyomyocytes start to lose the proliferation capacity [5]. In agreement with these reports, as shown in this study, the proliferation capacity of TnT-positive ES cell-derived cardiomyocytes dropped dramatically within a brief period from day 7.5 to day 10.5 of *in vitro* differentiation, which resulted in the small number of EdU-positive/Ki67-positive ES cell-derived cardiomyocytes in the control group at day 10.5. In this study, although we could not detect statistically significant differences between the Cited4 inhibition group and the control group due to this, the overexpression of the *Cited4* gene maintained the proliferation capacity of ES cell-derived cardiomyocytes expressing the myogenic marker of TnT. Moreover, at day 7.5, at which stage the majority of troponin T-positive ES cell-derived cardiomyocytes maintained the proliferation capacity as shown in this study, we confirmed that there was significant decrease of proliferation-competent TnT-positive ES cell-derived cardiomyocytes in the Cited4 inhibition group than in the control group. Furthermore, we confirmed no significant changes of the expression level of *Nav1*.*5* gene coding voltage-gated sodium channel, another marker for differentiation ES cell-derived cardiomyocytes [4], in the Cited4 overexpression group, compared with the control group. Taken together, it was suggested that the function of the *Cited4* gene was associated with the maintenance of proliferation capacity of TnT-positive ES cell-derived cardiac progenitor cells without inhibiting cardiomyocyte maturation. However, further studies are necessary to determine the possibility of the inhibitory funciton of the *Cited4* on cardiomyocyte maturation.

Some reports indicate that the *Cited4* gene contains domains that interact with several transcriptional factors or cofactors [1,18]. Therefore, the *Cited4* gene may have a number of mechanisms to control cell cycle-regulatory gene expression during *in vitro* cardiogenesis. Although the *C/EBPβ* gene is reported to relate to *Cited4* gene expression in adult mice [2], the precise regulatory mechanisms by which the *Cited4* gene controls cell cycle of cardiac progenitor cells are unknown. Further studies are needed to elucidate the functional roles of the *Cited4* gene in cell cycle of cardiac progenitor cells.

Cardiac regeneration is hampered by the low renewal rate of cardiomyocytes [19,20] or by the limited number of donor cardiomyocytes derived from ES cells [12]. Although the exogenous expression of *Myc* gene has been recently reported to promote the proliferation of cardiac progenitor cells, the intrinsic genes to control the proliferation capacity of cardiac progenitor cells are unknown [21]. Therefore, the intrinsic genes that enable cardiac progenitor cells to maintain proliferative capacity are of great interest. The results in this study indicated that the *Cited4* gene should be a candidate molecule for cardiac regeneration.

## Supporting information

S1 Protocol Western blot analysis of endogenous and exogenous Cited4 expression. The ht7 cell line without modification of the Cited4 gene expression was used as a control. The Cited4-ht7 cell line comprised cells with the overexpression of the FLAG-tagged Cited4 gene. The siCited4-ht7 cell line comprised cells with the knockdown of the Cited4 gene. Whole-cell lysates were collected using RIPA buffer from ES cells before differentiation at day 0 and EBs on day 6.5 after differentiation, and electrophoresed on 12% SDS-PAGE gel. The electrophoresed gels were transferred onto polyvinylidene fluoride membranes (Merck Millipore), and processed for Western blotting with an anti-Cited4, anti-FLAG tag, and anti-β-actin antibody in the same membrane: The membranes were blocked with 5% nonfat milk in TBST, incubated with diluted primary antibody overnight at 4°C, and then incubated with diluted secondary antibody for 1 h at room temperature. Antibodies are listed in S1 Table. Bands were visualized by chemiluminescent method using ECL plus system (Thermo Fisher Scientific).
(PDF)

S1 Table List of antibodies for Western blot analysis. (PDF)

S1 Fig Western blot analysis of endogenous and exogenous Cited4 expression. A. Analysis of endogenous and exogenous Cited4 expression levels with an anti-Cited4 antibody at day 0 and day 6.5. At day 0 of differentiation, the Cited4 expression was detected in the overexpression group, while it was hardly detected in the control and knockdown group. At day 6.5, the Cited4 expression level was increased 1.7-fold in the overexpression group and decreased 0.3-fold in the knockdown group, compared to the control group. B. Analysis of exogenous Cited4 expression levels with an anti-FLAG antibody at day 0 and day 6.5. Both at day 0 and day 6.5, the exogenous Cited4 expression was detected only in the overexpression group, but not in the control and knockdown group. C. Internal control for Western blotting. β-actin was used as an internal control for Western blotting.
(PDF)
