# Supplementary material for: Cited4 is related to cardiogenic induction and maintenance of proliferation capacity of embryonic stem cell-derived cardiomyocytes during in vitro cardiogenesis
Source: PLoS One. 2017 Aug 17;12(8):e0183225. doi: 10.1371/journal.pone.0183225 (PMC5560578; doi:10.1371/journal.pone.0183225)
Supplement: S1 Protocol — The ht7 cell line without modification of the Cited4 gene expression was used as a control. The Cited4-ht7 cell line comprised cells with the overexpression of the FLAG-tagged Cited4 gene. The siCited4-ht7 cell line comprised cells with the knockdown of the Cited4 gene. Whole-cell lysates were collected using RIPA buffer from ES cells before differentiation at day 0 and EBs on day 6.5 after differentiation, and electrophoresed on 12% SDS-PAGE gel. The electrophoresed gels were transferred onto polyvinylidene fluoride membranes (Merck Millipore), and processed for Western blotting with an anti-Cited4, anti-FLAG tag, and anti-β-actin antibody in the same membrane: The membranes were blocked with 5% nonfat milk in TBST, incubated with diluted primary antibody overnight at 4°C, and then incubated with diluted secondary antibody for 1 h at room temperature. Antibodies are listed in S1 Table. Bands were visualized by chemiluminescent method using ECL plus system (Thermo Fisher Scientific). (PDF) [file pone.0183225.s001.pdf]

S1 Protocol. Western blot analysis of endogenous and exogenous Cited4 expression.

The ht7 cell line without modification of the *Cited4* gene expression was used as a control. The Cited4-ht7 cell line comprised cells with the overexpression of the FLAG-tagged *Cited4* gene. The siCited4-ht7 cell line comprised cells with the knockdown of the *Cited4* gene. Whole-cell lysates were collected using RIPA buffer from ES cells before differentiation at day 0 and EBs on day 6.5 after differentiation, and electrophoresed on 12% SDS-PAGE gel. The electrophoresed gels were transferred onto polyvinylidene fluoride membranes (Merck Millipore), and processed for Western blotting with an anti-Cited4, anti-FLAG tag, and anti-β-actin antibody in the same membrane: The membranes were blocked with 5% nonfat milk in TBST, incubated with diluted primary antibody overnight at 4°C, and then incubated with diluted secondary antibody for 1 h at room temperature. Antibodies are listed in S1 Table. Bands were visualized by chemiluminescent method using ECL plus system (Thermo Fisher Scientific).
