# Supplementary material for: Cited4 is related to cardiogenic induction and maintenance of proliferation capacity of embryonic stem cell-derived cardiomyocytes during in vitro cardiogenesis
Source: PLoS One. 2017 Aug 17;12(8):e0183225. doi: 10.1371/journal.pone.0183225 (PMC5560578; doi:10.1371/journal.pone.0183225)
Supplement: S1 Table — (PDF) [file pone.0183225.s002.pdf]

**S1 Table. List of antibodies for Western blot analysis**

| Antibody | Dilution | Catalog Number | Company |
|---|---|---|---|
| **Anti-mouse polyclonal Cited4** | 1:1000 | ab105797 | Abcam |
| **Anti-monoclonal FLAG tag** | 1:1000 | 018-22381 | Wako |
| **HRP-conjugated anti-β-actin** | 1:5000 | Ab20272 | Abcam |
| **HRP-conjugated anti-rabbit IgG** | 1:3000 | NA934 | GE Healthcare |
| **HRP-conjugated anti-mouse IgG** | 1:3000 | NA931 | GE Healthcare |
