# Supplementary material for: Cited4 is related to cardiogenic induction and maintenance of proliferation capacity of embryonic stem cell-derived cardiomyocytes during in vitro cardiogenesis
Source: PLoS One. 2017 Aug 17;12(8):e0183225. doi: 10.1371/journal.pone.0183225 (PMC5560578; doi:10.1371/journal.pone.0183225)
Supplement: S1 Fig — A. Analysis of endogenous and exogenous Cited4 expression levels with an anti-Cited4 antibody at day 0 and day 6.5. At day 0 of differentiation, the Cited4 expression was detected in the overexpression group, while it was hardly detected in the control and knockdown group. At day 6.5, the Cited4 expression level was increased 1.7-fold in the overexpression group and decreased 0.3-fold in the knockdown group, compared to the control group. B. Analysis of exogenous Cited4 expression levels with an anti-FLAG antibody at day 0 and day 6.5. Both at day 0 and day 6.5, the exogenous Cited4 expression was detected only in the overexpression group, but not in the control and knockdown group. C. Internal control for Western blotting. β-actin was used as an internal control for Western blotting. (PDF) [file pone.0183225.s003.pdf]

S1 Fig.

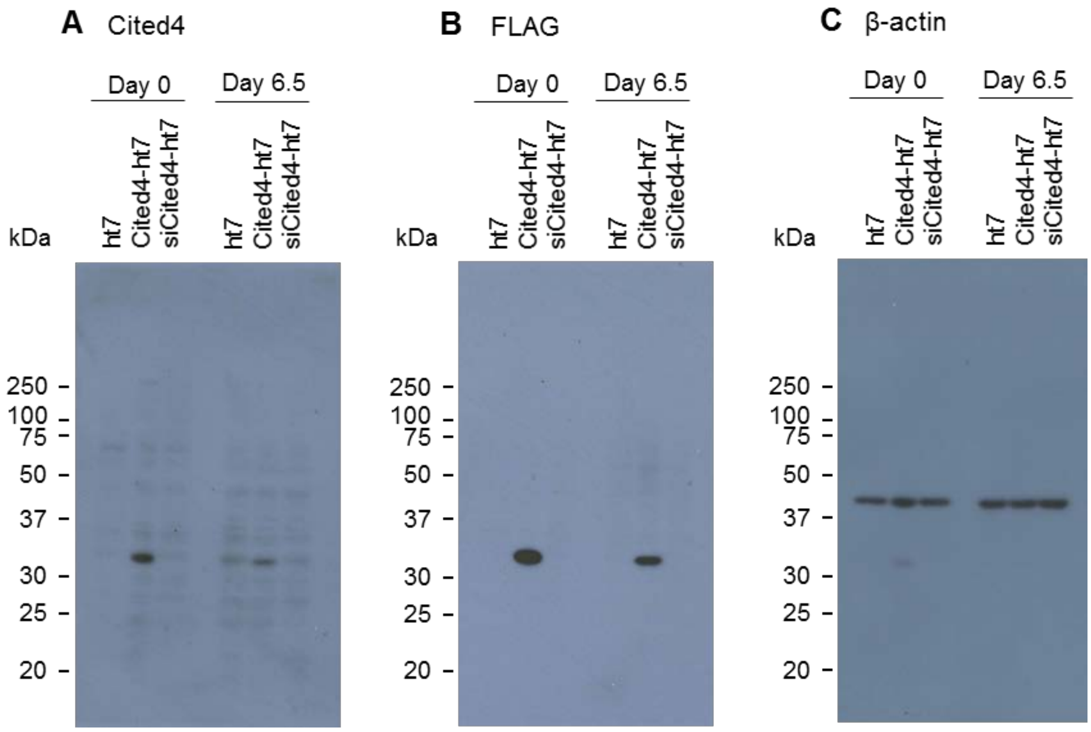

S1 Fig. Western blot analysis of endogenous and exogenous Cited4 expression.

A. Analysis of endogenous and exogenous Cited4 expression levels with an anti-Cited4 antibody at day 0 and day 6.5. At day 0 of differentiation, the Cited4 expression was detected in the overexpression group, while it was hardly detected in the control and knockdown group. At day 6.5, the Cited4 expression level was increased 1.7-fold in the overexpression group and decreased 0.3-fold in the knockdown group, compared to the control group. B. Analysis of exogenous Cited4 expression levels with an anti-FLAG antibody at day 0 and day 6.5. Both at day 0 and day 6.5, the exogenous Cited4 expression was detected only in the

overexpression group, but not in the control and knockdown group. C. Internal control for Western blotting. β-actin was used as an internal control for Western blotting.
